# Mechanical Impedance and Its Relations to Motor Control, Limb Dynamics, and Motion Biomechanics

**DOI:** 10.1007/s40846-015-0016-9

**Published:** 2015-01-27

**Authors:** Joseph Mizrahi

**Affiliations:** Department of Biomedical Engineering, Technion - Israel Institute of Technology, 32000 Haifa, Israel

**Keywords:** Mechanical impedance, Motor control, Motion biomechanics

## Abstract

The concept of mechanical impedance represents the interactive relationship between deformation kinematics and the resulting dynamics in human joints or limbs. A major component of impedance, stiffness, is defined as the ratio between the force change to the displacement change and is strongly related to muscle activation. The set of impedance components, including effective mass, inertia, damping, and stiffness, is important in determining the performance of the many tasks assigned to the limbs and in counteracting undesired effects of applied loads and disturbances. Specifically for the upper limb, impedance enables controlling manual tasks and reaching motions. In the lower limb, impedance is responsible for the transmission and attenuation of impact forces in tasks of repulsive loadings. This review presents an updated account of the works on mechanical impedance and its relations with motor control, limb dynamics, and motion biomechanics. Basic questions related to the linearity and nonlinearity of impedance and to the factors that affect mechanical impedance are treated with relevance to upper and lower limb functions, joint performance, trunk stability, and seating under dynamic conditions. Methods for the derivation of mechanical impedance, both those for within the system and material–structural approaches, are reviewed. For system approaches, special attention is given to methods aimed at revealing the correct and sufficient degree of nonlinearity of impedance. This is particularly relevant in the design of spring-based artificial legs and robotic arms. Finally, due to the intricate relation between impedance and muscle activity, methods for the explicit expression of impedance of contractile tissue are reviewed.

## Introduction: Redundancy, Mechanical Indeterminacy, and Lumping

The human motor system benefits from remarkable neuro-muscular redundancies. A motor task is normally performed with the concurrent involvement of more muscles than seemingly required. Further, a given task may be performed in multiple ways, each involving different motor units from several muscles that have to be orderly activated and coordinated. It has been demonstrated that the central nervous system makes use of simplifying strategies to facilitate complex tasks through the creation of muscle synergies that are put together by the motor cortical area and afferent systems [1]. From the mechanical viewpoint, the musculoskeletal system is indeterminate, with the number of unknown muscle forces exceeding the number of available equations [2].

The origin of neuro-muscular redundancy is expressed in the descending pathways from the central nervous system to the peripheral one. As a matter of fact, there exist multiple pathways for the execution of a given motor task such that the same information may be processed in different ways. Thus, a given central command may result in different activation combinations and, conversely, different commands can result in the same activation scheme. A trivial example arises when a joint torque of required intensity is to be provided. This torque can be produced in an infinite number of ways, involving co-contraction of the antagonist muscles at various activation intensities. From the purely mechanical aspect, the existence of co-contraction is actually undesirable because it results in a larger net joint force. Nonetheless, co-contraction is physiologically beneficial because it facilitates stability and controllability of posture and motion, sometimes at the cost of accelerated fatigue [3]. An interesting issue relates to the possibility to unequivocally resolve the muscle forces from the estimated joint torques. The redundancy of the musculoskeletal system provides numerous possible solutions to the problem of partitioning torque among active muscles [4, 5].

Thus, indeterminacy is associated with a multitude of possible solutions of the available system of equations. Conventional methods of addressing mechanical indeterminacy usually refer to the implementation of optimization criteria [6, 7], providing supplementary equations that allow eliminating irrelevant solutions. Nonetheless, the level of indeterminacy is expected to decrease with the reduction of redundancy [8]. In this respect, information about the activities of the muscles as expressed by their electromyograms (EMGs) may be instrumental in resolving muscle and joint forces and other unknowns from the musculoskeletal mechanical equations [2].

Indeterminacy of the locomotor system can also be addressed by implementing the lumping method, whereby the material elements of the human body, e.g., muscles, tendons, ligaments, bones, and joints, are lumped together in functional units so that the overall musculoskeletal system is represented as a one or more degrees-of-freedom (DOFs) damped elastic mechanism, interconnecting the masses of the body segments [9]. This method has been implemented for the modeling of various physical activities, including reaching motion for the upper limb [10–14] or, for the lower limbs, walking, hopping, and running. In this latter case, the foot- or heel-strike period during the landing phase has been modeled by means of one-dimensional lumped models (with linear displacements) or rotational lumping (with angular displacements) [15–23].

The idea of lumping has also been applied to the multi-segmental modeling of bilateral standing sway and standing imbalance [24–29] and the modeling of standing on one leg [4, 8].

The rest of this review is organized as follows. Section 2 presents the concept of mechanical impedance and the conditions where it may be treated as a constant or a variable property, thus giving rise to nonlinearities such as those in repulsive tasks or in human joints. Section 3 deals with the derivation of equations from which the mechanical impedance can be resolved and with methods of solutions of these equations. Section 4 lists the factors affecting mechanical impedance, including muscle activation, pre-programming of muscle activity, weight bearing, body configuration, types of interface between body and environment, nature of performed tasks, learning, and fatigue. Section 5 presents the intrinsic expression of mechanical impedance derived from the tissue level, as opposed to the system level, and expands this notion for the explicit expression of the impedance for contractile tissue. Finally, section 6 concludes the review by highlighting the relevance of mechanical impedance to control strategies of movement and the design of artificial legs and robots.

## Concept of Mechanical Impedance

### Definition

The complex relationship between deformation kinematics (generalized displacement/velocity) and the resulting dynamics (generalized force/torque), is termed mechanical impedance and defines the linear or rotational stiffness and damping characteristics of the system under consideration. The concept of impedance was first introduced in mechanical problems by Firestone [30]. In its simplest form, stiffness is defined as the ratio between the force change to the displacement change [31]. The impedance components (stiffness, effective mass, and damping) are particularly important for limb function since they counteract the effects of applied loads and disturbances.

In fact, a distinction should be made between impedance and admittance: while the first relates input kinematics to the resulting output dynamics of the system, the second relates the input dynamics to the resulting output kinematics. The electrical analogy of these two terms is with resistance and conductance which, like in mechanical systems, are also frequency-dependent. In linear systems, these two representations are usually equivalent and interchangeable. However, in nonlinear problems, such as those related to object manipulation, these two are not interchangeable [32]. For instance, for a hybrid controller implemented in the design of a torque-controlled manipulator to identify the dynamics of the wrist joint, the inertia and damping were implemented via admittance, and the stiffness via impedance [33].

### Average Impedance

A simpler definition of impedance makes reference to average stiffness, defined as the ratio between generalized force (force or torque) to overall generalized displacement (linear or angular) for the system under consideration (limb or joint). Similarly, average damping is obtained as the ratio between generalized force and generalized displacement rate.

In a review of spring–damper lumped systems, models were categorized as being either passive or active [34]. In passive models, the mechanical properties (stiffness and damping) were treated as being constant, and in active models, the mechanical properties were considered to adapt to external loads [34]. This categorization contradicts, however, other studies, which showed that nonlinearities of stiffness and damping can be found also in the passive state.

Past studies have modeled the human body using lumped components with constant average stiffnesses. Some examples are given below.

#### Vibrating Seats and Vehicles

This category includes multiple-DOF linear models of a seated vehicle driver [35–37], shipboard sitting subject to underwater shock [38], seated pregnant women for examination of the vibration effect on the woman and fetus under both horizontal and vertical vibrations [39, 40], and seated wheelchair users [41]. Using the electrical analogy method for solution, it was found that horizontal vibrations affect body segments more than do vertical vibrations [40].

With specific reference to seating design, an attempt was made to develop human body protection devices, such as cushions or seats with a shock absorber, in a vibration environment with a multiple-DOF lumped parameter model representing a seated human body/seat system and comprising the following two components: a global one, which included a systematic set of the model parameters for simulating various conditions such as body posture, backrest, footrest, muscle tension, and vibration directions; and a local one, which represented the human pelvis/vibrating seat contact, using a cushioning interface [42]. The very common seated position, particularly in the presence of vibrations, such as during the driving of a vehicle, is a subject of continuing interest as it is often related to the development of discomfort and back pain and spine disorders. Questions related to small–moderate variability of backrest inclination and backrest foam insertion, of position of the limbs while seated, and of the vibration conditions were shown to be satisfactorily addressed by a single-DOF lumped parameter model describing the vertical apparent mass of the human body. For example, contact with a rigid backrest, which is expected to reduce the vibrating mass and increase stiffness, resulted in an increase in the derived damped natural frequency of the principal resonance. An increase in the backrest inclination, which is also expected to reduce the moving mass, caused a decrease in the damping and an increase in the resonance frequency [43]. In search for criteria for developing active and passive isolation mechanisms for reducing the effects of whole-body vibration exposure while seated, one study developed a three-mass, two-DOF model that revealed nonlinear stiffness properties whereby the mean stiffness coefficients and the mean undamped natural frequencies associated with the upper torso and leg subsystems showed a significant decrease with increasing acceleration level [44].

#### Upper Limb

In the design of a torque-controlled manipulator, the wrist dynamics was described by a second-order inertia–spring–damper system and no distinction was made between muscle viscoelasticity and reflexive stiffness [33]. An apparatus was developed to quantify the average dynamic mechanical properties during active muscle exertions by delivering an external perturbation to the upper limb through a handle. The mechanical stiffness, damping, and effective mass elements were determined from free vibration displacement by calculating the frequency changes of the externally loaded system [45].

For the hand–arm system, early modeling with constant impedance made use of a two-DOF representation of the elbow–wrist joints with linear impedances to describe the dynamic response to vibration input to the hand and revealed that the hand–arm system may be viewed as a low-pass mechanical filter that attenuates high frequencies [12, 13]. A dynamic model of the biomechanics of the index finger including all the tendons and their varying moment arms assumed a constant stiffness [7]. Finger impedance measurements made under perturbations fast enough to prevent spinal reflex interference during data acquisition also assumed constant impedance [46].

#### Models for Physical Activities of Repulsive Tasks

These models have provided, to a limited extent, reasonable prediction of the foot–ground reaction forces while using lumped models with constant-average-stiffness elastic springs and negligible or no viscous damping [15, 21, 22, 47–50]. For running jumps for height and distance, segmental wobbling mass modeling was used to determine the elastic parameters of segments and the foot–ground interface [51].

#### Models for Carrying Backpacks

Lumped models simulating the interaction between the human trunk and a carried backpack using constant impedance values have been developed [52]. Backpack dynamics has been described using a nonlinear suspension model within whole body motion analysis during carrying. It was found that decreasing suspension stiffness moderated the peak values of vertical backpack force, acting on the trunk and lower limb joints. This reduced shoulder strap pressures and the risk of injury when heavy loads were carried. It was also found that the backpack suspension stiffness and damping have little effect on human locomotion energetics [53].

#### Spine Models

Numerous lumped models of the spine structure with constant impedance values have been developed. For example, a five-DOF lumped equivalent model with constant impedance values was developed for the lumbar spine [54], from which the postero-anterior motion could be predicted in the static, cyclic, and impulsive force modes.

### Variable Impedance

Linear models fail to predict the true dynamic response of the modeled systems. The use of invariable mechanical stiffness may not be physiologically applicable, as stiffness nonlinearity is important in stabilizing elastic chains during dynamic loading [55]. Joint impedance has thus been treated as a nonlinear phenomenon [19, 56–58].

It has been claimed that the determination of constant-value stiffness and damping coefficients is an ill-posed problem and that small errors in the measured kinematic data (i.e., noise, time delays) will lead to large errors in the estimated variables [59, 60]. It has thus been suggested that parameter estimation should rather be conducted using a moving-time-window algorithm, resulting in impedance values that are influenced not only by the nature of the perturbation but also by the time window over which the parameters are estimated [60]. A time-window method was implemented to determine the time-dependent changes in joint stiffness, damping, and equilibrium position in human forearm movements based on data of a single movement [61]. It should be noted, however, that although stiffness and damping in a stiffness–damping–inertia model is a nonlinear mixture of all the dynamic parameters of the musculoskeletal model, for practical purposes model representation of the impedance redundancies can be reduced to less cumbersome nonlinearities [14].

#### Physical Activities of Repulsive Tasks

##### Hopping

Repulsive tasks such as human hopping, jumping, and running were studied by assuming position-dependent and/or angle-dependent joint stiffnesses in three-segment models [55]. Damping, though generally not negligible, was found to be low. More specifically, for hopping motion, rotational springs with nonlinear stiffnesses in a four-segment leg model revealed that the correct and sufficient variability of the joint stiffness is of first order (i.e., linearly variable with joint angle) [19].

Muscular activation level, which in hopping motion determines the ability of the muscle–tendon complex to absorb, store, release, and generate energy, varies during the stance phase. Since the stiffness generally depends on the activation level of the muscle [3, 62], it follows that hopping cannot be treated as a purely harmonic motion and that human joints are not simple mechanical springs.

##### Running

For the stance phase of human running, the leg was modeled as a one-dimensional four-DOF lumped system [9]. Departing from the constant-stiffness concept, the model revealed that a correct and sufficient variability of the joint stiffness is a two-region piece-wise constant joint stiffness, indicating that a higher order of nonlinearity is unnecessary. This result should be considered meaningful for problems where the constant stiffness representation is insufficient and in cases where the system’s representation has to be improved. As already mentioned, joint stiffness is dominated by muscular activation [63, 64] and as the joints stiffen, they experience a decrease in angular displacements during the ground-contact phase, resulting in smaller excursion of the hip and higher leg stiffness. Solutions obtained using piece-wise constant stiffness provide, through the obtained stiffness profiles, an insight into the patterns of the muscular activation in the legs’ joints. A simple model with a piece-wise constant stiffness can predict major features of running, making it an effective tool for the design of artificial legs and robots and also for the development of more accurate control strategies.

It was also reported that during heel strike, the joints did not have a damping effect, and thus did not contribute to energy dissipation [9].

#### Ankle Joint

The complexity of the ankle joint presents an interesting challenge for modeling and resolving mechanical impedance. Considering first the mechanics of this joint in the sagittal plane, i.e., with the motion taking place in plantar and dorsi flexion of the joint, the relationship between the applied external moment and the resulting angular displacement can be studied in oscillatory motion, in which the foot is driven relative to the shank with the torque being applied by means of a band-limited oscillatory Gaussian signal [65]. This setting also allows studying the effects on mechanical impedance of the activation level of the muscles around the joint, and of the dynamic reflex as a result of applying a sudden torque. Variations in the resting posture of the tested subject, the mode of attachment of the foot, and the manner of applying the displacement perturbations to the foot were later introduced to this method [66]. The amplitude of motion and fatigue of the muscles during exercise were found to be of considerable significance to mechanical impedance values.

Further, by applying stochastic perturbation of the ankle angular position for actively exerting plantar or dorsi flexion torques, mechanical impedance was evaluated in the ankle joint for a range of muscle activation levels. It was found that while the elastic stiffness proportionally increased with the mean torque at all levels of muscle activation, damping remained almost invariant over the entire range of contractions [64].

The above studies about the ankle joint in plantar/dorsi flexion motion made use of a second-order quasi-linear underdamped system, by which elastic stiffness, damping coefficient, natural frequency, and moment of inertia of the foot about the ankle joint could be evaluated. Stiffness and damping characteristics of the ankle joint plantar flexors were not affected by static stretching [67, 68].

For the subtalar joint, statically evaluating the stiffness of various football boots in inversion–eversion motion revealed that when using rigidly attached high boots, ligamentous load on the subtalar joint was reduced considerably, indicating conditions under which footwear may protect the joint [60]. For that same joint, for an unexpected and sudden inversion motion of the foot, it was reported that the stretch reflex on the peroneal muscles remains unelicited for a period of approximately 70 ms from the onset of motion [69]. Subsequently, the dynamic properties of the human subtalar joint in sudden inversion motion, which is likely to occur in conditions of inversion sprain of the ankle joint, were studied in vivo on a specially designed apparatus [70]. This joint was modeled as a quasi-linear second-order underdamped system, from which the elastic stiffness and damping coefficient, the natural frequency of the foot, and its moment of inertia about the joint were evaluated. The variation of mechanical impedance with joint angle was confirmed [70].

Stiffness, damping, and inertia parameters were also studied in three- and four-DOF models of the lower limb in torsion, in which a dynamic pulse loading was applied at the foot–boot level [71, 72]. For rotation motion, the dynamics of the ankle in the medial–lateral plane in both laboratory and skiing apparatuses was studied to identify stiffness, damping, and inertia parameters [72]. Weight bearing on the foot and muscle-induced torsion were used as test variables, and were found to be important in studying the dynamics of the ankle. Comparing this model with a more simplified single-DOF model, which best duplicates the rotation of the knee, indicated that the latter model gave good prediction of knee rotation, with stiffness symmetrical with respect to the rotation direction [71].

#### Upper Limb

The mechanical impedance of the upper limb is of great interest because of the many tasks assigned to the upper limb, including manual tasks (such as grasping and/or using hand and power tools) and reaching motions. Prior to a detailed discussion on these tasks, it should be mentioned that torsional pendulum tests were implemented on the upper limb, allowing limb inertia and muscle stiffness to be calculated [73].

##### Grasping Objects and Reaching Motion

Numerous studies have modeled grasping and reaching motion. With the objective to map the field of restoring forces associated with posture in the horizontal plane of the multiarticular arm, small displacements along various directions were delivered to the hand [10]. Measured force and displacement vectors before the onset of voluntary reaction were used to estimate the stiffness in the vicinity of the hand equilibrium position. It was found that the forces were predominantly conservative, indicating a spring-like behavior of the neuromuscular system. A defined stiffness ellipse, representing the main geometrical features of the elastic force field, was defined as follows: the major and minor axes were scaled to the maximum and minimum stiffness, respectively; the ellipse area was scaled to the total stiffness, and the orientation of the ellipse was made to coincide with the direction of the major (or minor) axis. Interestingly, the obtained ellipse field was found to be invariant among the tested subjects and over time of testing. Further, when a disturbance acting along a fixed and predictable direction was imposed, the magnitude of the total stiffness increased but only minor changes in shape and orientation occurred, shedding light on neural factors involved in maintaining hand posture [10]. Measuring the postural force field of a subject’s arm over relatively large distances, and comparing these forces with the static forces generated at the hand while the subject attempted a reaching movement, confirmed the hypothesis that the central nervous system programs a reaching movement by shifting the equilibrium position of the hand toward the target [74]. However, due to the large displacements from equilibrium, the use of a nonlinear model (i.e., a variable stiffness matrix) is required to describe the measured postural field [74].

As later shown, during simple point-to-point movement, the human arm did not behave in a conservative manner and could not even be described as a stable passive dynamic system around a desired trajectory. In fact, muscle properties and automatic reflex response can result in significant deviations from the desired trajectory, with over-compensation in the form of an active response [75].

##### Grasping Objects and Locomotion

An interesting problem presents when grasping takes place simultaneously with locomotion, requiring the coordination of these two modes of control [14, 76–78]. More specifically, the effect of constraint of the joints of the upper limb was studied to shed light on the mechanisms of stabilization of manually held objects during walking through impedance adjustments [14]. This was performed by successively immobilizing each of the joints while subjects walked steadily with a hand-held cup filled with liquid. The ability to maintain the liquid level was used to indicate how well the limb navigated itself to minimize liquid spillage from the cup and how the limb adapted to the imposed joint restrictions. This problem has strong relevance in impedance-based control strategies and provides an insight into the mechanisms by which stiffness and damping are adjusted to accommodate changes taking place during manual transport of objects while aiming to ensure their stability during walking.

A two-dimensional sagittal model consisting of three segments, namely upper arm, forearm, and hand connected together by the joints via lumped parameter impedances representing damped springs, was thus set to describe the system. The semi-repetitive oscillations of the shoulder girdle produced by the ambulatory mechanism in concert with the motion of the joints of the upper limb navigated the hand holding the cup of liquid in an oscillatory-like motion. With the gait-generated motion of the shoulder girdle and limb serving as inputs to the model and the kinematics of the hand holding the fluid filled cup serving as output, the impedance adjustments were resolved during the simultaneous control of grasping and walking under ordinary conditions, and when each one of the joints is immobilized.

#### Neck Modeling in Impact Motion

A lumped model to describe neck behavior in low-velocity rear-end impact implemented on a dummy satisfactorily predicted the response when elastic stiffness and damping were combined in the muscle substitutes with a nonlinear stiffness of model joints [79].

## Derivation of Equations for Dynamic Model

### Nature of Driving Perturbation

Impedance values can be generally derived from the dynamic equations of motion, through which relationships between the driving perturbation and the resulting response can be obtained. Methods can be grouped according to the mode of applied driving force or displacement perturbation as follows.

#### Oscillatory Methods

A common method to resolve mechanical impedance from oscillatory motion was implemented in the wrist joint [80] and ankle joint [65]. In this latter case, the foot was driven relative to the shank and the torque was delivered by means of a band-limited oscillatory Gaussian signal. This provided the relationship between the applied external moment and the resulting angular displacement of the joint. Oscillatory methods were also used to study the biomechanical characteristics of the muscles of the human ankle joint [81] and the human arm [82, 83].

#### Impact Perturbation

A fast forward head movement in the frontal plane was modeled to study head–neck joint response [84]. If the perturbation is fast enough, the effect of the accompanied muscular activity can be neutralized. A method to accomplish that was devised for inversion motion of the subtalar joint by suddenly and unexpectedly rotating the foot relative to the shank on a specially constructed swiveling platform, driven by stretched springs. If motion lasts less than 40 ms, the protective muscles are not elicited by the stretch reflex [69]. The impedance properties obtained from this experiment represent the passive properties of the ankle joint in inversion/eversion motion in sprain-like conditions of the ankle. Using appropriate instrumentation, information about torque and kinematics can be obtained [70]. The same idea of preventing the stretch reflex by sufficiently fast perturbations was applied for measuring human finger impedance [46].

#### Pendulum Oscillations

Oscillatory testing of the lower leg about the knee joint was implemented for deriving stiffness and damping of the joint muscles, i.e., for the extensors in vibration mode [85, 86], a pendulum test [87–89], and joint flexors [90]. The validity of the pendulum motion test for testing spasticity around the knee joint has been studied for two cases [91]: (a) gravity-induced free oscillations in which no reflex excitation occurred, and (b) spastic limb oscillations in which reflex excitation did occur. In the first case, a linear second-order model was not adequate to satisfactorily describe the motion and asymmetries in the amplitude dependencies had to be the incorporated in the impedance parameters. In the second case, EMG-based impedance components had to be added [91]. Similarly, for the elbow, a linear second-order model for pendulum testing indicated that the stiffness coefficient remained relatively consistent among the groups tested. In testing spasticity, both the damping coefficient and damping ratio increased in the affected side of stroke patients and tended to increase with the severity of spasticity [92]. Pendulum passive testing of the elbow joint revealed that the obtained impedance properties were similar between men and women with comparable body weights and did not deteriorate significantly with age [93]. Torsional pendulum tests were also implemented on the upper limb, allowing limb inertia and muscle stiffness to be calculated [73].

#### Static, Dynamic, and Creep Tests

Static, dynamic, and creep tests of the human knee in vivo were performed to estimate the viscoelastic properties of the knee [94]. The dynamic disturbance was provided by means of a sinusoidal displacement generator to excite the knee–leg system and the driving force was monitored as a function of the frequency and mechanical characteristics of the knee. It was found that in quasi-static loading, the knee exhibited hysteresis. In dynamic loading, the knee behaved as a single-DOF spring–mass–damper system with a St. Venant’s friction element [94].

#### Common Daily Activities

Other methods used for studying how the system reacts to perturbations made use of common ordinary daily activities. This can be illustrated in locomotion modeled as an inverted pendulum with springs and damping, through which various effects of impairments such as spasticity or muscle weakness in cerebral palsy can be estimated [95]. Another example for a natural task is found in the design of torque-controlled manipulator (with torque disturbances) to identify the dynamics of the wrist joint, both under natural and neurologically impaired conditions, while the subject actively controls the joint angle [33]. Other activities were used for evaluating the joint impedance of the lower limbs: (a) simulation of single-leg standing using an open-loop model [4], (b) swaying motion in bi-pedal standing, used to feed a three-dimensional five-segment model with four joints by means of which an iterative estimation of the kinematics and dynamics of the system was conducted, opening the possibility of the estimation of the mechanical impedances in the joints of the lower extremity, power transfer through the joints, and the production of muscle forces [26], (c) hopping motions using a four-link lumped model [19], (d) jumping and running motion to establish the input force for one-dimensional multi-DOF impedances [9, 15, 35, 37, 81, 96], and (e) seated postures in oscillatory environments [35–43, 97]. Upper limb models were utilized for the simulation of various tasks, such as grasping during semi-oscillatory walking motion [14].

### Governing Equations

The dynamics of interconnected lumped segments can be expressed using Newton–Euler equations of motion [15, 26, 59, 70] and inverse-dynamics methods, such as for impact loading of the lower leg [9, 19] or step loading of the elbow [98]. Alternatively, velocity-based inverse-dynamics equations using Kane’s [14, 99] method can be used. For single-stance standing, open-chain inverse-dynamics was used [4, 100, 101]. The governing equations for expressing the mechanical impedances in lumped systems can be categorized as follows.

#### Second-Order System

Second-order quasi-linear underdamped systems have been used for oscillatory motion [65, 66], impact motion [15, 70], and isotonic loading of the trunk [62] to evaluate elastic stiffness, damping coefficient, natural frequency, and moment of inertia. Such systems were implemented for the ankle joint in plantar and dorsi flexion motion [65, 66], the wrist joint [80], and the trunk using the load sudden-release method [102]. Active extension exertions were also used in the trunk to estimate the active impedance component values using second-order trunk dynamics following preload that caused small amplitude trunk movements [103].

#### Regressive Function

Regressive functions can be used to express stiffness and damping in terms of joint angle and joint angular velocity. For instance, it has been found that for the upper limb joints during grasping control taking place simultaneously with walking motion, stiffness includes first-order dependence on angle and angular velocity. The function used for damping included first-order dependence on angular velocity [14]. For the lower limb, it was found that during hopping motion, stiffness included second-order dependence on angle and first-order dependence on angular velocity and that damping was negligible [19]. The logic behind representing such a regressive function stems from the fact that the mechanical properties of a biological material can, in general, be multiple-variable-dependent. Specifically, stiffness, in addition to being nonlinear (e.g., strain-dependent), may often depend on the deformation rate. This is the case with bones [104], tendons and ligaments [105], cartilage [106], and muscle [107]. Similarly, damping can be position-dependent. Accordingly, stiffness and damping during the stance phase of hopping can be represented by a general second-order regressive function.

#### Additional Methods

Alternative methods include using an artificial neural network for the prediction of the biodynamic response during vertical vibration of the seated human body in a sitting posture without a backrest [97].

### Methods of Solution

The stiffness and damping coefficients can be resolved from the governing equations by parameter estimation using optimization procedures such as minimization of an objective function for each of the joints.

The model parameters should be independent of each other and, if multiple linear regression analyses are performed, for the parameter estimation to be correct all predictor variables must be uncorrelated. In the numerical solution procedure, it is thus necessary to reveal dependencies and eliminate redundancies of the stiffness and damping coefficients. This may be done using multicollinearity diagnostic algorithms combined with *F*-test [108]. Parameter identification can be performed using either quadratic programming [109] or a genetic algorithm [110]. Two examples are given below.

#### Hopping Motion

In hopping motion, after eliminating redundancies in the numerical solution using the above-mentioned multicollinearity diagnostic algorithms, the model revealed that the correct and sufficient variability of the joint stiffness is of first order, indicating that a higher order of nonlinearity is not necessary [19]. This result should be considered meaningful in problems where a constant stiffness representation is insufficient and in cases where the system’s representation has to be improved. The variable stiffness solution also provides, through the obtained stiffness profiles, an insight into the patterns of the muscular activation in the legs’ joints.

The fact that the simple model of a linearly variable stiffness can predict major features of the jumping exercise makes it an effective tool for future design of artificial legs and robots and also for the development of more accurate control strategies.

#### Simultaneous Grasping and Locomotion

The results obtained in the cup of liquid problem, in which the spring coefficients were expressed in terms of the joint angles and angular velocities, indicated a continuous nonlinear behavior of the joints. This sheds light on the design of spring-based artificial and robotic arms [14]. The wrist joint was found to have constant stiffness and damping and thus no regulation of these coefficients was necessary during gait. This is consistent with results for moderately sized wrist rotations, indicating that a simple, linear model can be used for studies in biomechanics, motor neuroscience, and rehabilitation, neglecting nonlinearities [111]. The passive stiffness of the wrist is sufficient to account for the pattern of the path of rotation [112]. Dominance of the passive viscoelastic (particularly the stiffness component) torques in planar wrist movements was also confirmed [113]. These findings somewhat contradict earlier ones, indicating that while the muscle properties around the wrist were basically spring-like, there are major nonlinearities in the viscoelastic properties of the wrist [114]. Testing the wrist using the vibration method revealed that the stiffness, viscosity, and damping ratio decreased significantly across all displacements, with higher values for small angles and lower values with increasing flexion [80].

Both in the elbow and shoulder joints, stiffness included a constant coefficient as well as an angular-velocity-dependent coefficient, with no damping. These results confirmed that, in this case, a higher order of nonlinearity was not necessary.

## Factors that Affect Mechanical Impedance

The ability to tune and/or control the mechanical impedance of the limb joints is an important feature of the neuromuscular system [32, 115] and of multi-joint powered orthotics [116]. Tuning of the mechanical impedance facilitates, for example, the stabilization of hand-held objects in space, or the attenuation of undesired shock loads resulting from externally applied forces [11, 46, 117].

Mechanical impedance may generally depend upon numerous factors, including muscle activation, load or weight bearing, loading conditions, position or posture of the system (such as the joint angle), interface properties between the human body and the contacting surface, task, learning and training, and physiological conditions such as wellness, fitness, fatigue, and possible existence of various pathologies. Examples of the latter include recurrent low back pain, in which trunk stiffness was found to be higher and damping lower compared to the normal case [102], contrary to recurrent ankle sprain in which balance was not found to be impaired [27].

Low back pain recurrence was also found to be related with lower stride-to-stride variability of trunk motion, resulting from a protective movement strategy, possibly based on increased trunk stiffness and damping [118].

### Muscle Activation

It has been suggested that, mechanically, a muscle is analogous to a spring, whose stiffness is a function of its activation. As with a spring, a muscle’s force is a function of its length [119]. It should be noted that in the limb joints, impedance can also be adjusted by the activation level of the antagonist muscles of the joint. Thus, for a required net torque in the upper limb, different levels of contraction of the antagonists will result in different impedance levels and, accordingly, in different energy costs of that net torque [115, 120].

For the lower extremity, a high correlation was found between pre-contact EMG activity and the EMG activity in the concentric phase (brake phase) of the ground-contact period [121]. It was also reported that muscles can reduce the vertical peak ground reaction force due to their ability to absorb energy during impact [122], and that recruitment and activation of stiffness proportional to initial stiffness can be achieved by positive muscle force feedback [123]. It should be remembered, though, that repetitive performance of a task may fatigue the active mechanisms of the human body [124].

It has also been found that in relation to minimizing soft-tissue vibrations, such as when energy dissipation is not desirable, impedance tuning is achieved by adjusting muscle activity in reaction to impact forces [125]. Prior to landing, muscle activity is responsible for generating the correct joint stiffness as determined by co-contraction of the muscles surrounding the joint [126]. In general, the central nervous system uses different mechanisms to tune the mechanical properties of the muscles to the different required tasks [127].

For the body trunk, to empirically evaluate the influence of coactivation on trunk stiffness, trunk dynamics, including stiffness, mass, and damping, were quantified during trunk extension exertions with and without voluntary recruitment of antagonistic co-contraction. It was reported that co-contraction increases trunk stiffness, thereby supporting the idea that co-contraction may contribute to spinal stability [128].

#### Impedance Tuning Through Pre-programmed Non-reflex Action: Pre-activation

Several factors have been reported to affect reduction of peak forces as a result of landing impact.

In repulsive tasks, impedance tuning through pre-programmed non-reflex muscle action during the early phase of impact helps reduce peak forces. The necessity of setting the joint angles and of tuning the stiffness before leg loading was reported [129]. In hopping exercise, the presence of an initial joint stiffness was found, suggesting that muscle pre-activation is important in controlling the peak forces [19]. It should be noted, however, that no direct information was provided about which leg muscles are part of this activity. For that purpose, EMG measurements should be taken in synchrony with the measured kinematics and foot–ground reaction forces. In a model that included wobbling masses to simulate the impact force peaks during running, it was shown that tuning of the muscle activation of the lower extremities alongside with changes of joint angles and joint angular velocities could result in controlling the impact force [130].

The pre-programmed non-reflex muscle action during the early phase of impact has been proven to be important in peak force attenuation. This procedure can be trained for and controlled by the subject to achieve simultaneous use of all joints and coordination between the various segments of the leg. Better attenuation is also a result of increasing the flexion range of the joints of the leg [131].

When deformation starts in passive mechanisms, a neurological feedback system senses the resulting increased force and so brings muscles into play before the forces have had time to reach destructive levels [132]. The ability to pre-program muscle action and joint motion is thus of major importance when reflex activity has not yet appeared.

It should be noted, however, that the paraspinal reflexes have been shown to augment effective stiffness [120]. An increased reflex gain was reported following prolonged trunk flexion, which in turn may contribute to low back pain risk [133]. In fact, it has been shown that intrinsic muscle stiffness alone is insufficient for stability and that reflex dynamics are necessary components in the stabilizing control of spinal stability [134].

In the elbow and wrist joints, using a model with linearly time-varying angular stiffness and viscosity, it has been observed that anticipatory muscle stiffening and anticipatory flexion of the limb are synergistic in building up resistance of the hand to a catch task and that reflex coactivation produces a further increment of hand stiffness and viscosity to counteract the impact effect [135].

It should be noted that reflex mechanisms can be fatigued, as may happen with individuals performing repetitive tasks [124].

### Weight or Load Bearing

It has been reported that the magnitude of the force acting on the leg affects its stiffness [20]. Weight bearing on the foot was also found to influence mechanical impedance in torsion testing of the lower leg [72]. In a large-scale pulse rotation test of the lower leg in which joint rotation was studied, it was noted that joint stiffness increased somewhat with weight bearing [71]. Increasing load was also found to increase the overall stiffness and damping coefficients in the subtalar joint [70].

### Conditions of Dynamic Loading

Impedance dependency with weight bearing, direction of perturbation, and displacement was reported in a large-scale pulse rotation test of the lower leg in which joint rotation was studied [71]. Specifically, the dependency of mechanical impedance on perturbation frequency has been the subject of numerous investigations. For instance, the variation of dynamic stiffness and damping of the vertebral discs in the range of frequencies transmitted by a car seat has been reported [136]. To simulate shock loading taking place during running, a multi-DOF lumped model of a standing human body was subjected to impulse inputs at the foot [96]. When comparing linear and nonlinear simulations, a significant decrement was found in the acceleration transfer magnitude in the nonlinear model and nonlinear damping was found to be significant. Subjects with higher body fat could have a greater degree of nonlinear damping, which provides better attenuation and higher energy dissipation [96].

The loading rate of the impact force at heel strike, as applied by a pendulum on the foot, was found to affect muscle activation, as measured by EMG of the lower leg muscles [137].

### Effect of Body Configuration

In standing posture, it has been shown that stiffness of the leg decreases with increasing knee flexion and is maximal in a fully extended knee [20]. In the trunk, conversely, stiffness was reported to increase with flexion angle [133].

In impacting activities, among the factors that were reported to affect peak force attenuation as a result of landing impact, initial flexion of the joints plays an important role [131]. During landing impact, high forces are imposed on the leg with the result that, if a low stiffness of the leg is desired (to reduce impact forces), a relatively high initial flexion angle of the joints is required. This, however, would considerably limit the range (amplitude) of flexion in these joints afterwards to effectively absorb the energy by the muscles spanning the joints. This latter point was quantitatively studied by evaluating the amount of elastic energy which could be stored and re-used in human hopping. It was concluded that the dissipated energy in muscles increased when the amplitudes of joint movements were larger [138]. Utilization of stored elastic energy was reported to depend on the shortness in latency between the stretch and shortening phases of the muscles [139]. If one is interested in dissipation of energy, such as during landing from a height, the bigger the range of joint angle, the better. Presentation of the muscle as an energy dissipating shock absorber as studied on the cat soleus revealed two prominent damping nonlinearities: motion amplitude and movement history dependencies [140].

Multi-joint flexion in activities of repulsive tasks is probably the basis for reducing peak forces at impact [131]. In this case, proper coordination between the different joints is important. Increasing task complexity, as may be presented in multi-joint action, was shown to decrease efficiency [141]. The reason for this is related to timing in multi-joint motion, which affects movement synchronization of the various segments of the leg. For the overall performance to be fully efficient, the acceleration maxima of the different segments should be in phase. It should be noted that correct action of the joints and muscles can achieve this effect even before the reflex activity has come into action.

Another example related to multi-joint coordination relates to sit-to-stand transition motion. Particularly, in people with pathology such as Parkinson’s disease, slowness of a sit-to-stand transition could be due to a reduced hip flexion joint torque and a prolonged rate of torque production. This could point to impairments in the ability to control sequential and/or coordinated movements of the joints [142–145]. It remains unsettled, however, whether motor deficits observed in subjects with Parkinson’s disease during chair-rise tasks are related to the lower limbs torques, and whether muscle weakness and rate of force generation impair the ability to tune the joint impedances.

For the upper limb joints, stiffness regulation was also reported to depend upon posture configuration [14, 146, 147]. One study on the upper limbs, with actively maintained elbow angles, showed that both the stiffness and viscosity increase with increasing elbow flexion [148]. In reaching motion, however, the value of hand stiffness depends not only on the joint angular stiffnesses but also on the geometrical configuration that the limb takes in space at any instant [10]. Rotational stiffness and damping were measured for the elbow extensor muscle. Stiffness was also found to depend on gender, elbow flexion angle, and co-contraction level while damping depended on the latter only [98].

In studying the relation between posture and reaching movement, equilibrium of the upper limb is assumed to be reached at a position at which the length-dependent forces due to the opposing activations of the agonist and antagonist muscles are equal [119]. If this position is considered as an equilibrium position, it was hypothesized that reaching movements can be regarded as shifts in equilibrium positions [74]. This, however, somewhat contradicts earlier results about the relationship between movement amplitude and stiffness of the forearm movement that indicated that trajectory formation was more complex than a simple switch between equilibrium points [149].

### Interface Between Body and Contacting Surface

Other factors affecting mechanical impedance include the body/support interface, such as footwear and terrain (e.g., ground stiffness) [150]. Static evaluation of the stiffness of various football boots in inversion–eversion motion showed that when using rigidly attached high boots, ligamentous load on the subtalar joint is reduced considerably [151]. The effect of footwear protection was pronounced in the elastic stiffness parameter in testing of the subtalar joint under sudden inversion motion [70]. Footwear was also indicated to be important in conjunction with speed when considering resonant vibration in soft tissue; it was reported to have a significant contribution to the energy dissipation after an impact [125].

Soft landing in jumping motion for the attenuation of peak force depends on the quality of the ground and of the shoes worn by the subject. A distinction should be made between voluntary landing and hopping motions; in the former, the muscle is transformed from a spring to a damping unit, intended to absorb and dissipate energy, whereas in the latter, the muscle stiffness remains positive [127]. Thus, foam-rubber sheets on the landing surface can be expected to decrease the stiffness of the impact medium and thus reduce impact [131]. A study on the optimization of landing mat properties found that damping was far more influential in peak force reduction compared to stiffness [152]. The attenuation effect can be attributed not only to a decrease in stiffness but also to an increase in contact time during impact. For a given linear impulse, a contact time increase results in a decrease of the impulsive forces present. In one study on human running, it was demonstrated that a more compliant track increased the contact time of the foot with the ground [18].

Soft landing can also be achieved by landing on the balls of the feet to make use of the cushioning effect of the foot heel-pad tissue. The balls of the feet have a lower stiffness compared to that of the flat part of the foot [9]. Additionally, landing on the ball of the foot allows increasing the flexion range during impact. Landing on the balls of the feet considerably decreased peak forces as compared to landing with the feet flat [131]. Also here, another contributing factor is the contact time; the longer it is, the smaller peak are the forces likely to result.

For a one-DOF inverted pendulum model representing the colliding leg in running, the natural frequency of the cushioning mechanism was estimated using linearized and extended Kalman filter estimators [153]. In this model, the stiffness and damping of the foot-surface cushion represented the fat pad layer at the bottom of the heel and the running surface. This stiffness is directly proportional to the estimated natural frequency. Thus, it has been suggested that leg stiffness is not directly related to running mechanics, but, rather, to the running environment [153]. In gait, the increased effective leg stiffness with speed supports the greater propulsion energy required before foot contact during faster gaits [31].

Testing of the coupling of footwear and the supporting ground confirmed that ground stiffness strongly affects the impact forces and that it should therefore be considered as an essential parameter in footwear design [154]. Ground stiffness and damping were also reported to influence hopping strategies through adjustment of the spring-like mechanics of the leg and surface combination to regulate the body center of mass and work output during exercise [155].

The dynamics of impact to the hip during a fall was studied during pelvis release experiments in which the dynamic response of the body to a step change in vertical force applied to the hip was measured. The simplest mathematical model capable of simulating the problem consisted of a single effective mass attached to three sets of spring–damper elements. The effective moving mass was located at the hip and one vertical spring–damper combination represented the structural properties of the skin, fat, and muscle within the contact area, as well as the compressive properties of the proximal femur, hip joint, and pelvis. The remaining elements were two horizontally oriented elements that consisted of the combined flexural stiffness and damping of the muscles and ligaments that span the spine and connect the pelvis to the trunk and lower limbs. These elements constrained the hips and pelvis from lateral excursions from the midline of the body [156].

For the upper extremities, the reduction of impact forces during forward falls onto outstretched hands was analyzed using linear models with surface stiffness modification. It was found that at the moment of impact, compliant surfaces attenuate the high-frequency peak force, which affects the wrist by decreasing the velocity across the wrist damping elements. At the same time, the lower-frequency peak deflection of the shoulder spring is not substantially reduced [157].

### Task Dependency

The dynamic behavior of the motor system is considerably different from static behavior (where muscle stiffness behaves uniformly and consistently as springs) and it adapts to the requirements of the motor task [127]. For instance, during voluntary landing, the muscle is transformed from a spring to a damping unit, intended to absorb and dissipate energy. While muscle stiffness in the ankle joint is negative after touch down when landing, it always remains positive when hopping [127].

Dual tasking, combining postural control (inverted pendulum model) with outside distraction, leads to decreased stiffness and increased sway amplitude in the mediolateral direction by diverting the resources necessary for mediolateral postural control, thus increasing the risk to falls [158]. It should be mentioned in this respect that an increased dispersion of sway in the mediolateral plane has also been associated with impaired vision and fall risk in the elderly [159]. This group modeled postural sway during standing to retrieve the viscoelastic parameters of swaying motion using two methods: second-order oscillatory model and discrete second-order autoregressive model.

Impedance values were thus reported to depend on task constraints [14, 160–164], the patterns of perturbation, and the actual joint movement configuration [10, 146, 165, 166]. Accordingly, in defining impedance characteristics, stiffness and damping coefficients have been mostly assumed to be variable [61, 167] with resulting dependencies upon deformation and rate of deformation [14].

In long-distance running, for instance, moderate speed may result in more than 300 foot strikes/leg/km. Each such foot strike evokes an impact loading that results in a vertical shock impulse transmitted upwards through the body and carries with it the potential for injuries in the bone and joint tissues. Fatigue or stress fractures occur in bones in response to repetitive stresses over multiple cycles when the body’s ability to adapt is exceeded [150, 168]. An important factor that affects the incidence of bone stress injury is exposure to abrupt changes in the bone loading [150] and consequent alteration in the strain distribution [169] with insufficient recovery periods [170]. Within the mechanical impedance parameters of the lower limbs, a major factor responsible for impulse attenuation at foot or heel strike is the shock absorption capacity of the active muscle in the lower limbs. Additionally, impact forces initiate vibrations of the wobbling soft tissues during the landing phase of running [171, 172]. The vibrations of these non-rigid masses, particularly those of the lower body, affect impact forces [172] and soft tissue vibrations and have been reported to contribute significantly to the energy dissipation after an impact [125, 173].

During a running task, it was assumed that the properties of the model remain unchanged and the effect of mass and shoe hardness on the ground reaction forces was studied [172]. A modification on this model introduced a shoe-specific nonlinear function for representation of the ground reaction force, resulting in a better agreement between simulation and experimental results [174]. Later, by hypothesizing that the central nervous system keeps the level of the vibrations of the human body constant using muscle tuning, it has been shown that a wobbling mass model can correctly simulate the effects of shoe hardness on the vibrations of the human body during running [175].

### Learning and Training

The level of preparation of movement coordination depends on the training stage of the subject [141]. Training should not only prepare the subject for optimal use of the joint ranges but also to reduce the time difference between the segmental acceleration maxima to zero. Training was also reported to augment muscle stiffness [90].

The possible effects of learning over the time of conducting the experiments beyond the warm-up phase should be considered. Learning was reported to decrease joint total stiffness [11]. While the training effect on stiffness was reported to be dependent on the training intensity [150], when the training level was similar to the exercise level, learning usually took place within the first few sessions of training [176] and then leveled off, i.e., the difference between the test means in two consecutive sessions was not statistically significant.

### Fatigue

Fatigue of the muscles affects not only their performance but other related functions as well such as unsteadiness of performance [66]. It has been shown [177] that muscles act to lower the bending stress on bone and to attenuate the peak dynamic loads that can damage musculoskeletal tissues. Muscle fatigue has also been shown to affect the ability of the human musculoskeletal system to attenuate and dissipate the heel strike induced shock waves (for running [178], for long-march [179]). Some studies [180–182] have suggested that when the muscle’s ability to perform is diminished, the cartilage and ligaments become more vulnerable to excess dynamic loading, which in turn may increase stiffness. Nevertheless, it has been claimed that, in direct contrast to experiments on whole body fatigue, localized muscle fatigue was found to cause a decrease in peak tibial acceleration and acceleration slope following impact [183].

In addition to muscle fatigue, metabolic fatigue may take place when the running subject exceeds a running speed termed the anaerobic threshold [184, 185]. With progressing fatigue in long distance level running, the effect on mechanical impedance can be inferred from the fact that the impact shock load on the lower limbs increases [186–189]. One additional question is whether, as a result of fatigue, an imbalance between the activities of the plantar and dorsi flexor muscles of the ankle develops. Such an imbalance would further compromise the protective action provided by the muscles to the shank [190–192].

Measurements of the foot-strike-evoked impact at both the tibial tuberosity and sacrum levels by means of accelerometers demonstrated an increase of the impact in both locations with developing fatigue, indicating a diminished capacity to attenuate the foot-strike-initiated shock waves [189]. Time and frequency domain analyses of the acceleration data suggested that fatigue contributes to the reduction of the human musculoskeletal system’s capacity to attenuate and dissipate those shock waves. This capacity appears to be a function not only of the fatigue level, but also of the vertical location along the skeleton. It seems that higher up the skeletal, parts are able to withstand the effect of fatigue for a longer time.

In decline running, metabolic fatigue is less likely to develop compared to level running due to the reduced effort required for running. However, due to their eccentric activity, the major knee extensors do get fatigued, resulting in significantly increased shock acceleration at the sacrum level, while the shock acceleration at the tibial tuberosity remains unchanged [186]. Thus, without metabolic fatigue development, shock propagation from the tibial tuberosity to the sacrum is augmented due to the eccentric action of the muscles.

Apart from the impact shock transmission, reported to provoke bone injury and joint damage, stride rate (frequency) at a given speed (i.e., stride length) was identified to affect stiffness. It was argued that a stiffer leg leads to a higher stride frequency and shorter stride length at a given speed [21, 22, 47–49]. Interactions between stiffness and kinematics were also noted, indicating that the impact shock can be attenuated by adjusting the joint stiffness and/or the joint kinematics, thus providing support for the concept of muscle tuning during dynamic activities and suggesting that speed/shoe combinations are important when considering resonant vibration in soft tissue [125, 193, 194].

## Material Evaluation of Impedance (vs. System or Structural)

By direct in vivo and in vitro measurement of tissue segments, the passive viscoelastic parameters can be obtained. From quasi-static in vitro mechanical testing of vertebral bodies from the thoracic spine, flexibility and stiffness matrices from the load–displacement diagrams and the variation of the mechanical properties with the spine level were obtained [195]. In vivo creep experiments of lumbar motion segment in centric passive tension during the course of hydrotherapy, using ultrasound for deformation measurements, revealed that stiffness and damping of these elements were gender- and age-dependent [196].

Passive tests on the knee joint based on the free damped oscillation technique indicated that the passive viscoelastic properties were in a small range of values and invariant during the growth process of this body segment. Accordingly, the passive viscoelastic properties cannot be held responsible for abnormal control in human spastic paresis and cannot be used as a descriptor of spasticity [197].

Impedance of soft tissue (e.g., on limb surfaces) or hard tissue (e.g., forehead) has been measured by means of a loading device using the random force vibration method [198]. These investigators, however, commented on the difficulty of tissue impedance measurements because this impedance is small compared to the impedance of the measuring device.

### Explicit Expression of Impedance of Contractile Tissue

To study the contribution to mechanical impedance of the muscle–tendon component by means of micro-structure-based phenomenological modeling, most early muscle models were used for investigating short term-tasks, such as distance jumping, high jumping, pedaling, kicking, and gait [199], typically lasting less than 2 s, with small interference of fatigue. More general muscle models should, however, include long-term dynamic features such as the force build-up within the muscle and fatigue effects.

Muscle and muscle–tendon system models have been proposed by several investigators [200–202], with the effects of the inner structure of the system explicitly expressed. In these models, though, an inherent difficulty is encountered due to the multitude of physiological parameters required. Some of these parameters are functional, e.g., rheological, describing the stress–strain relationships of the muscles and tendons [203–206]. These types of parameters are referred to as muscle nonspecific parameters. The other types of parameters describe individual characteristics of the particular muscles modeled. They are associated mostly with the anthropometric measures of the individual muscles such as size, cross-sectional area, and mass of the muscles and tendons and are termed muscle-specific parameters.

A Huxley-type muscle–tendon model consisting of five elements was formulated and formed the basis of subsequent work [200]. This model included the tendon serial elastic element and four additional elements pertaining to the muscle itself, namely the parallel elastic element [207], the contractile element [208], the damper element [209, 210], and the muscle mass. Despite the functional distinction characterized in the model, it should be noted that interactive effects may be present, such that stretching of the muscle–tendon may evoke a reduction in the viscosity of the muscle [211]. The mentioned Huxley-type model was implemented for expressing the human forearm system [212–215] and the human thigh [216, 217]. It has provided improvement of the dynamic performance of the human intact forearm by simulating tendon transfer [212]. A further extension of the improved upper limb model allowed comparing the dynamic performances of amputated forearms, with and without prosthesis, in various structural configurations. Amputation variables were represented by the modified anthropometry, modes of reinsertion of the residual muscles, and possible flapping of the muscle around the stump [214].

The above five-element muscle–tendon model was also incorporated in the lower limb to enable long-term prediction of the force output of the quadriceps muscle during continuous electrical stimulation. In the contractile element, muscle activation was explicitly expressed by means of the EMG signal while muscle fatigue was incorporated by using a specially introduced term based on the concentration of intracellular pH [216–219]. The presence of fatigue during prolonged FES causes a substantial decrease in the force output of the quadriceps muscle [220]. The metabolic parameters recorded using magnetic resonance spectroscopy [221] revealed fatigue profiles (parallel to the force profile) when measured during stimulation and recovery profiles when measured after stimulation. These profiles served for prediction of the dynamic force in intermittent stimulation.

## Conclusion

Tuning of the mechanical impedance is a major factor in asserting the sound performance of the many tasks assigned to the limbs and in counteracting undesired effects of applied loads and disturbances. It facilitates the control and stabilization of hand-held objects in space and their navigation in reaching motions as well as the attenuation of undesired shock loads resulting from externally applied forces. The ability to tune and/or control the mechanical impedance of the limb joints is not only an important feature of the neuromuscular system, but has also strong relevance in the design of torque-controlled manipulators and multijoint powered orthotics. In early works, impedance was conveniently assumed to be constant, resulting in linear models. However, more recent work has repeatedly demonstrated that linear models fail to predict the true dynamic response of modeled systems and that the use of constant mechanical impedance may not physiologically be applicable. Impedance nonlinearity is therefore required. An important reason is that impedance is intricately related to the mode and amount of muscle activation involved in the performance of as given task. It should be noted however that while estimating the nonlinear impedance component values, care should be taken to eliminate redundancies by reducing the model to reveal the correct and sufficient degree of impedance nonlinearity. For instance, in modeling grasping simultaneously taking place with locomotion, the spring coefficients should be expressed in terms of the joint angles and angular velocities, indicated a continuous nonlinear behavior of the joints. Both in the elbow and shoulder joints, stiffness includes a constant coefficient as well as an angular-velocity-dependent coefficient, with no damping. These results confirmed that a higher order of nonlinearity was not necessary. This result should be considered meaningful in problems where the constant stiffness representation is insufficient and in cases where the system’s representation has to be improved. This problem has strong relevance in impedance-based control strategies and provides insight into the mechanisms by which stiffness and damping are adjusted to accommodate changes taking place during simultaneous walking and manual transportation of objects, while aiming to ensure their stability. It also sheds light on the design of spring-based artificial and robotic arms. Another example is in tasks of repulsive loads, where the nonlinearity of the impedance of the leg joints is found to be expressible by means of a simple model, with a linearly variable stiffness. This enables the prediction of the major features of the jumping exercise, making it an effective tool for the future design of spring-based artificial legs and robots and the development of more accurate control strategies.

## References

[1] Bizzi, E., & Cheung, V. C. K. (2013). The neural origin of muscle synergies. *Frontiers in Computational Neuroscience,**7*, Article 51. doi:10.3389/fncom.2013.00051. Published: 29 April 2013. 10.3389/fncom.2013.00051 PMC3638124 23641212

[2] Mizrahi, J. (2011). The role of electromyograms in resolving musculoskeletal interactions in able-bodied and disabled human individuals. In J. Mizrahi (Ed.), *Advances in applied electromyography* (pp. 3–24). Rijeka: InTech. ISBN 978-953-307-382-8.

[3] Gardner-Morse MG, Stokes IA (1998). The effects of abdominal muscle coactivation on lumbar spine stability. Spine.

[4] Mizrahi J, Brion O, Adam D (2002). Open-chain analysis of single stance. Journal of Automatic Control.

[5] Levin, O., Mizrahi, J., Adam, D., Verbitsky, O., & Isakov, E. (2000). On the correlation between force plate data and EMG in various standing conditions. In T. Sinkjaer, D. Popovic & J. J. Struijk (Eds.), *Proceedings**of the fifth annual conference of the International Functional Electrical Stimulation**Society*, 18–24 June (pp. 47–50). Aalborg: Center for Sensory-Motor Interaction, Aalborg University.

[6] Patriarco AG, Mann RW, Simon SR, Mansour JM (1981). An evaluation of the approaches of optimization models in the prediction of muscle forces during human gait. Journal of Biomechanics.

[7] Brook N, Mizrahi J, Shoham M, Dayan J (1995). A biomechanical model of index finger dynamics. Medical Engineering and Physics.

[8] Suponitsky Y, Verbitsky O, Peled E, Mizrahi J (2008). Effect of force imbalance of the shank muscles, due to selective fatiguing, on single-leg-standing control. Journal of Electromyography and Kinesiology.

[9] Mizrahi, J., & Daily, D. (2012). Modeling the foot-strike event in running fatigue via mechanical impedances. In T. Goswani (Ed.), *Injury and skeletal biomechanics* (pp. 153–170). Rijeka: InTech. ISBN 978-953-51-0690-6.

[10] Mussa-Ivaldi F, Hogan N, Bizzi E (1985). Neural, mechanical, and geometric factors subserving arm posture in humans. Journal of Neuroscience.

[11] Stroeve S (1999). Impedance characteristics of neuromusculoskeletal model of the human arm, I. Posture control. Biological Cybernetics.

[12] Rosenbaum DA, Meulenbroek RG, Vaughan J, Jansen C (2001). Posture-based motion planning: Applications to grasping. Psychological Review.

[13] Suggs CW, Taylor W (1974). Modelling of the dynamic characteristic of the hand–arm system. The vibration syndrome.

[14] Roth N, Seliktar R, Mizrahi J (2011). Mechanical impedance control in the human arm while manually transporting an open-top fluid filled dish. Applied Bionics and Biomechanics.

[15] Mizrahi J, Susak Z (1982). Elastic and damping response of the human leg to in vivo impact forces. Journal of Biomechanical Engineering: Transactions of the ASME.

[16] Ozguven HN, Berme N (1988). An experimental and analytical study of impact forces during human jumping. Journal of Biomechanics.

[17] Kim W, Voloshin AS, Johnson SH (1994). Modeling of heel strike transients during running. Human Movement Science.

[18] McMahon TA, Green PR (1979). The influence of track compliance on running. Journal of Biomechanics.

[19] Rapoport S, Mizrahi J, Kimmel E, Verbitsky O, Isakov E (2003). Constant and variable impedance of the leg joints in human hopping. Journal of Biomechanical Engineering: Transactions of the ASME.

[20] Greene PR, McMahon TA (1979). Reflex stiffness of man’s anti-gravity muscles during kneebends while carrying extra weights. Journal of Biomechanics.

[21] Farley CT, Gonzalez O (1996). Leg stiffness and stride frequency in human running. Journal of Biomechanics.

[22] Farley CT, Morgenroth DC (1999). Leg stiffness primarily depends on ankle stiffness during human hopping. Journal of Biomechanics.

[23] Spagele T, Kistner A, Gollhofer A (1999). Modeling, simulation and optimization of a human vertical jump. Journal of Biomechanical Engineering: Transactions of the ASME.

[24] Mizrahi J, Dvir Z (2000). Biomechanics of balance. Clinical biomechanics.

[25] Levin O, Mizrahi J (1996). An iterative model for estimation of the trajectory of center of gravity from bi-lateral reactive force measurements in standing sway. Gait Posture.

[26] Levin O, Mizrahi J, Shoham M (1998). Standing sway: Iterative estimation of the kinematics and dynamics of the lower extremities from forceplate measurements. Biological Cybernetics.

[27] Isakov E, Mizrahi J (1997). Is balance impaired by recurrent sprained ankle?. British Journal of Sports Medicine.

[28] Isakov E, Mizrahi J (1997). Bilateral simultaneous measurement of standing ground reaction forces in hemiparetics, below-knee amputees and healthy adults. Basic and Applied Myology.

[29] Isakov E, Mendelevich I, Ring H, Mizrahi J (1998). Balance recovery pattern in recent hemiplegics. Europa Medicophysica.

[30] Firestone, F. A. (1938). The mobility and classical impedance analyses. In *American Institute of Physics handbook* (pp. 3–140). New York: McGraw-Hill Book Company.

[31] Kim S, Park S (2011). Leg stiffness increases with speed to modulate gait frequency and propulsion energy. Journal of Biomechanics.

[32] Hogan N (1985). Impedance control: An approach to manipulation: Parts I, II and III. Journal of Dynamic Systems Measurement and Control: Transactions of the ASME.

[33] Schouten AC, de Vlugt E, van Hilten JJ, van der Helm FC (2006). Design of a torque-controlled manipulator to analyse the admittance of the wrist joint. Journal of Neuroscience Methods.

[34] Nikooyan A, Zadpoor AA (2011). Mass–spring–damper modelling of the human body to study running and hopping—An overview. The Proceedings of the Institution of Mechanical Engineers, Part H: Journal of Engineering in Medicine.

[35] Amirouche FML (1987). Modeling of human reactions to whole body vibrations. Journal of Biomechanical Engineering: Transactions of the ASME.

[36] Boileau PE, Rakheja S (1998). Whole-body vertical biodynamic response characteristics of the seated vehicle driver: Measurement and model development. International Journal of Industrial Ergonomics.

[37] Patil MK, Palanichamy MS, Ghista DN (1980). Response of human body to tractor vibrations and its minimization by provision of relaxation suspensions to both wheels and seat at the plane of centre of gravity. Medical and Biological Engineering and Computing.

[38] Zong Z, Lam KY (2002). Biodynamic response of shipboard sitting subject to ship shock motion. Journal of Biomechanics.

[39] Qassem W (1996). Model prediction of vibration effects on human subject seated on various cushions. Medical Engineering and Physics.

[40] Qassem W, Othman MO (1996). Vibration effects on setting pregnant woven-subjects of various masses. Journal of Biomechanics.

[41] Pavec D, Aubin CE, Aissaoui R, Parent F, Dansereau J (2001). Kinematic modeling for the assessment of wheelchair user’s stability. IEEE Transactions on Neural Systems and Rehabilitation Engineering.

[42] Rosen J, Arcan M (2003). Modeling the human body/seat system in a vibration environment. Journal of Biomechanical Engineering: Transactions of the ASME.

[43] Toward MG, Griffin MJ (2010). A variable parameter single degree-of-freedom model for predicting the effects of sitting posture and vibration magnitude on the vertical apparent mass of the human body. Industrial Health.

[44] Smith SD, Kazarian LE (1994). The effects of acceleration on the mechanical impedance response of a primate model exposed to sinusoidal vibration. Annals of Biomedical Engineering.

[45] Sesto ME, Radwin RG, Richard TG (2005). Short-term changes in upper extremity dynamic mechanical response parameters following power hand tool use. Ergonomics.

[46] Fiorilla E, Nori F, Masia L, Sandini G (2011). Finger impedance evaluation by means of hand exoskeleton. Annals of Biomedical Engineering.

[47] Arampatzis A, Bruggemann G-P, Metzler V (1999). The effect of speed on leg stiffness and joint kinetics in human running. Journal of Biomechanics.

[48] Farley CT, Blickhan R, Saito J, Taylor CR (1991). Hopping frequency in humans: Test of how springs set stride frequency in bouncing gaits. Journal of Applied Physiology.

[49] Farley CT, Houdijk HHP, van Strien C, Lourie M (1998). Mechanism of leg stiffness adjustment for hopping on surfaces of different stiffnesses. Journal of Applied Physiology.

[50] McMahon TA, Cheng GC (1990). The mechanics of running—How does stiffness couple with speed?. Journal of Biomechanics.

[51] Wilson C, King MA, Yeadon MR (2006). Determination of subject-specific model parameters for visco-elastic elements. Journal of Biomechanics.

[52] Foissac M, Millet GY, Geyssant A, Freychat P, Belli A (2009). Characterization of the mechanical properties of backpacks and their influence on the energetic of walking. Journal of Biomechanics.

[53] Ren L, Jones RK, Howard D (2005). Dynamic analysis of load carriage biomechanics during level walking. Journal of Biomechanics.

[54] Keller TS, Colloca CJ (2002). A rigid body model of the dynamic posteroanterior motion response of the human lumbar spine. Journal of Manipulative and Physiological Therapeutics.

[55] Seyfarth A, Gunther M, Blickhan R (2001). Stable operation of an elastic three-segment leg. Biological Cybernetics.

[56] Hayes KC, Hatze H (1977). Passive viscoelastic parameters of the structures spanning the human elbow joint. European Journal of Applied Physiology.

[57] Karniel A, Inbar GF (1999). The use of nonlinear muscle model in explaining the relationship between duration, amplitude, and peak velocity of human rapid movements. Journal of Motor Behavior.

[58] Rakheja S, Gurram R, Gouw GJ (1993). Development of linear and nonlinear hand–arm vibration models using optimization and linearization techniques. Journal of Biomechanics.

[59] Kalveram K Th (1991). Pattern generating and reflex-like processes controlling aiming movements in the presence of inertia, damping and gravity. A theoretical note. Biological Cybernetics.

[60] Kistemaker DA, Rozendaal LA (2011). In vivo dynamics of the musculoskeletal system cannot be adequately described using a stiffness–damping–inertia model. PLoS ONE.

[61] Konczak J, Brommann K, Kalveram KT (1999). Identification of time-varying stiffness, damping, and equilibrium position in human forearm movements. Motor Control.

[62] Granata KP, Rogers E (2007). Torso flexion modulates stiffness and reflex response. Journal of Electromyography and Kinesiology.

[63] Nielsen, J., Bisgard, C., Arendt-Nielsen, L., & Jensen, T. S. (1994). Quantification of cerebellar ataxia in movements of the hand. In *Biomechanics*, seminar 8, Goteburg, Sweden (pp. 157–166).

[64] Weiss PL, Hunter IW, Kearney RE (1988). Human ankle joint stiffness over the full range of muscle activation levels. Journal of Biomechanics.

[65] Gottlieb GL, Agarwal GC (1978). Dependence of human ankle compliance on joint angle. Journal of Biomechanics.

[66] Kearney RE, Hunter IW (1982). Dynamics of human ankle stiffness: Variation with displacement amplitude. Journal of Biomechanics.

[67] Hunter DG, Coveney V, Spriggs J (2001). Investigation into the effect of static stretching on the active stiffness and damping characteristics of the ankle joint plantar flexors. Physical Therapy in Sport.

[68] Knudson D (2006). The biomechanics of stretching. Journal of Exercise Science and Physiotherapy.

[69] Isakov E, Mizrahi J, Solzi P, Susak Z (1986). Response of the peroneal muscles to sudden inversion of the ankle during standing. International Journal of Sport Biomechanics.

[70] Mizrahi J, Ramot Y, Susak Z (1990). The passive dynamics of the subtalar joint in sudden inversion of the foot. Journal of Biomechanical Engineering: Transactions of the ASME.

[71] Johnson C, Hull ML (1988). Parameter identification of the human lower limb under dynamic, transient torsional loading. Journal of Biomechanics.

[72] Mote D, Lee CW (1982). Identification of human lower extremity dynamics in torsion. Journal of Biomechanics.

[73] Walsh EG, Wright GW (1987). Inertia, resonant frequency, stiffness and kinetic energy of the human forearm. Quarterly Journal of Experimental Physiology.

[74] Shadmehr R, Mussa-lvaldi FA, Bizzi E (1993). Postural force fields of the human arm and their role in generating multijoint movements. Journal of Neuroscience.

[75] Popescu, C., & Rymer, Z. (1996). Is the human arm made of tunable springs? In *18th annual international conference of the IEEE Engineering in Medicine and Biology Society*, Amsterdam (pp. 587–588).

[76] Carnahan H, McFadyen BJ, Cockell DL, Halverson AH (1996). The combined control of locomotion and prehension. Neuroscience Research Communications.

[77] Georgopoulos AP, Grillner S (1989). Visuomotor coordination in reaching and locomotion. Science.

[78] Van der Wel RPRD, Rosenbaum DA (2007). Coordination of locomotion and prehension. Experimental Brain Research.

[79] Linder A (2000). A new mathematical neck model for a low-velocity rear-end impact dummy: Evaluation of components influencing head kinematics. Accident Analysis and Prevention.

[80] Halaki M, O’Dwyer N, Cathers I (2006). Systematic nonlinear relations between displacement amplitude and joint mechanics at the human wrist. Journal of Biomechanics.

[81] Aruin S, Zatsiorsky VM (1984). Biomechanical characteristics of human ankle-joint muscles. European Journal of Applied Physiology and Occupational Physiology.

[82] von Gierke HE, Coermann RR (1963). The biodynamics of human response to vibration and impact. Industrial Medicine and Surgery.

[83] Hondori HM, Shih-Fu L (2010). Perturbation-based measurement of real and imaginary parts of human arm’s mechanical impedance. Conference Proceedings of the IEEE Engineering in Medicine and Biology Society.

[84] Pedrocchi A, Ferrigno G (2004). Model of head–neck joint fast movements in the frontal plane. Biological Cybernetics.

[85] Crowninshield AR, Pope MH, Johnson R, Miller R (1976). The impedance of the human knee. Journal of Biomechanics.

[86] Casabona A, Valle MS, Pisasale M, Pantò MR, Cioni M (2012). Functional assessments of the knee joint biomechanics by using pendulum test in adults with Down syndrome. Journal of Applied Physiology.

[87] Bajd T, Vodovnik L (1984). Pendulum testing of spasticity. Journal of Biomedical Engineering.

[88] Fee JW, Miller F (2004). The Leg Drop Pendulum Test performed under general anesthesia in spastic cerebral palsy. Developmental Medicine and Child Neurology.

[89] Oatis CA (1993). The use of a mechanical model to describe the stiffness and damping characteristics of the knee joint in healthy adults. Physical Therapy.

[90] Blackburn JT, Riemann BL, Padua DA, Guskiewicz KM (2004). Sex comparison of extensibility, passive, and active stiffness of the knee flexors. Clinical Biomechanics.

[91] Lin C, Rymer WZ (1991). A quantitative analysis of pendular motion of the lower leg in spastic human subjects. IEEE Transactions on Biomedical Engineering.

[92] Lin CC, Ju MS, Lin CW (2003). The pendulum test for evaluating spasticity of the elbow joint. Archives of Physical Medicine and Rehabilitation.

[93] Lin C, Ju MS, Huang HW (2005). Gender and age effects on elbow joint stiffness in healthy subjects. Archives of Physical Medicine and Rehabilitation.

[94] Pope MH, Crowninshield R, Miller R, Johnson R (1976). The static and dynamic behavior of the human knee in vivo. Journal of Biomechanics.

[95] Fonseca ST, Holt KG, Saltzman E, Fetters L (2001). A dynamical model of locomotion in spastic hemiplegic cerebral palsy: Influence of walking speed. Clinical Biomechanics.

[96] Jarrah M, Qassem W, Othman M, Gdeisat M (1997). Human body model response to mechanical impulse. Medical Engineering and Physics.

[97] Abdeen MAM, Abbas W (2011). Prediction the biodynamic response of the seated human body using artificial intelligence technique. International Journal of Engineering.

[98] Lee Y, Ashton-Miller JA (2011). The effects of gender, level of co-contraction, and initial angle on elbow extensor muscle stiffness and damping under a step increase in elbow flexion moment. Annals of Biomedical Engineering.

[99] Kane TR, Levinson DA (1985). Dynamics: Theory and application.

[100] Asada H, Slotine JJE (1986). Robot analysis and control.

[101] Denavit J, Hartenberg RS (1955). A kinematic notation for lower pair mechanisms based on matrices. Journal of Applied Mechanics.

[102] Hodges P, van den Hoorn W, Dawson A, Cholewicki J (2009). Changes in the mechanical properties of the trunk in low back pain may be associated with recurrence. Journal of Biomechanics.

[103] Moorhouse KM, Granata KP (2005). Trunk stiffness and dynamics during active extension exertions. Journal of Biomechanics.

[104] Wright TM, Hayes WC (1980). Tensile testing of bone over a wide range of strain rates: Effects of strain rate, micro-structure and density. Medical and Biological Engineering and Computing.

[105] Peterson, R. H., Gomez, M. A., & Woo, S. L.-Y. (1987). The effects of strain rate on the biomechanical properties of the medial collateral ligament: A study of immature and mature rabbits. In *Transactions of the 33rd annual meeting of the Orthopaedic Research Society* (Vol. 12, p. 127). 10.1002/jor.1100080513 2388111

[106] Li, J. T., Armstrong, C. G., & Mow, V. C. (1983). The effects of strain rate on mechanical properties of articular cartilage in tension. In S. L.-Y. Woo & R. Mates (Eds.), *Proceedings of the biomechanical symposium ASME AMD* (Vol. 56, pp. 117–120).

[107] Herzog W, Leonard TR (1991). Validation of optimization models that estimate the forces exerted by synergistic muscles. Journal of Biomechanics.

[108] Slinker BK, Stanton AG (1985). Multiple regression for physiological data analysis: The problem of multicollinearity. American Journal of Physiology.

[109] Gill PE, Murray W, Wright MH (1981). Practical optimization.

[110] Davis L (1991). Handbook of genetic algorithms.

[111] Charles SK, Hogan N (2011). Dynamics of wrist rotations. Journal of Biomechanics.

[112] Charles SK, Hogan N (2012). Stiffness, not inertial coupling, determines path curvature of wrist motions. Journal of Neurophysiology.

[113] Deshpande D, Gialias N, Matsuoka Y (2012). Contributions of intrinsic visco-elastic torques during planar index finger and wrist movements. IEEE Transactions on Biomedical Engineering.

[114] Gielen C, Houk JC, Marcus SL, Miller LE (1984). Viscoelastic properties of the wrist motor servo in man. Annals of Biomedical Engineering.

[115] Hogan N, Winters JM, Woo SL-Y (1990). Mechanical impedance of single- and multiarticular systems. Multiple muscle systems: Biomechanics and movement organization.

[116] Lemay MA, Hogan N, van Dorsten JW (1998). Issues in impedance selection and input devices for multijoint powered orthotics. IEEE Transactions on Rehabilitation Engineering.

[117] Volpe, R., & Khosla, P. (1990). Theoretical analysis and experimental verification of a manipulator/sensor/environment model for force control. In *Proceedings IEEE international conference on systems*, *man and cybernetics*, 4–7 November 1990 (pp. 784–790).

[118] van den Hoorn W, Bruijn SM, Meijer OG, Hodges PW, van Dieën JH (2012). Mechanical coupling between transverse plane pelvis and thorax rotations during gait is higher in people with low back pain. Journal of Biomechanics.

[119] Feldman AG (1966). Functional tuning of the nervous system during control of movement or maintenance of a steady posture. III. Mechanographic analysis of the execution by man of the simplest motor tasks. Biophysics.

[120] Franklin TC, Granata KP (2007). Role of reflex gain and reflex delay in spinal stability—A dynamic simulation. Journal of Biomechanics.

[121] Aura O, Viitasalo JT (1989). Biomechanical characteristics of jumping. Journal of Applied Biomechanics.

[122] Gerritsen GM, Bogert AJ, Nigg B (1995). Direct dynamics simulation of the impact phase in heel-toe running. Journal of Biomechanics.

[123] Geyer H, Seyfarth A, Blickhan R, Muller R (2001). Proprioceptive feedback in running. Proceedings of the XVIII congress of the International Society of Biomechanics.

[124] Radin, E. L. (1974). Nature of mechanical factors causing degeneration of joints. In Hip, *proceedings of the 2nd open scientific meeting of the Hip Society* 1974 (pp. 76–81). St. Louis, MO: C.V. Mosby.

[125] Boyer KA, Nigg BM (2004). Muscle activity in the leg is tuned in response to impact force characteristics. Journal of Biomechanics.

[126] Boyer KA, Nigg BM (2007). Changes in muscle activity in response to different impact forces affect soft tissue compartment mechanical properties. Journal of Biomechanical Engineering: Transactions of the ASME.

[127] Dyhre-Poulsen P, Simonsen EB, Voigt M (1991). Dynamic control of muscle stiffness and H reflex modulation during hopping and jumping in man. Journal of Physiology.

[128] Lee PJ, Rogers EL, Granata KP (2006). Active trunk stiffness increases with co-contraction. Journal of Electromyography and Kinesiology.

[129] Golhofer A, Strojnik V, Rapp W, Schweizer L (1992). Behavior of triceps surae muscle–tendon complex in different jumping conditions. European Journal of Applied Physiology.

[130] Nigg BM, Liu W (1999). The effect of muscle stiffness and damping on simulated impact force peaks during running. Journal of Biomechanics.

[131] Mizrahi J, Susak Z (1982). Analysis of parameters affecting impact force attenuation in landing of human vertical free fall. Engineering in Medicine.

[132] Finlay JB, Repo RU (1979). Energy absorbing ability of articular cartilage during impact. Medical and Biological Engineering and Computing.

[133] Granata KP, Rogers E, Moorhouse K (2005). Effects of static flexion-relaxation on paraspinal reflex behavior. Clinical Biomechanics.

[134] Moorhouse KM, Granata KP (2007). Role of reflex dynamics in spinal stability: Intrinsic muscle stiffness alone is insufficient for stability. Journal of Biomechanics.

[135] Lacquaniti F, Maioli C (1989). The role of preparation in tuning anticipatory and reflex responses during catching. Journal of Neuroscience.

[136] Izambert O, Mitton D, Thourot M, Lavaste F (2003). Dynamic stiffness and damping of human intervertebral disc using axial oscillatory displacement under a free mass system. European Spine Journal.

[137] Wakeling JM, Von Tscharner V, Nigg BM, Stergiou P (2001). Muscle activity in the leg is tuned in response to ground reaction forces. Journal of Applied Physiology.

[138] Thys H (1978). Evaluations indirecte de l’energie elastique utilisee dans l’impulsion des sauts. Schweiz Z. Sportmed..

[139] Bosco C, Komi PV (1979). Mechanical characteristics and fiber composition of human leg extensor muscles. European Journal of Applied Physiology.

[140] Lin, C., & Rymer, W. Z. (1997). Nonlinear damping properties and postural stability of the neuromuscular system. In *Proceedings of the 19th international conference IEEE*/*EMBS*, Chicago, IL, USA, 1652–1655, 30 October–2 November 1997.

[141] Luhtanen P, Komi PV (1978). Segmental contribution to forces in vertical jump. European Journal of Applied Physiology.

[142] Nikfekr E, Kerr K, Attfield S, Playford DE (2002). Trunk movements in Parkinson’s disease during rising from seated position. Movement Disorders.

[143] Seidler RD, Alberts JL, Stelmach GE (2001). Multijoint movement control in Parkinson’s disease. Experimental Brain Research.

[144] Serrien DJ, Steyvers M, Debaere F, Stelmach GE, Swinnen SP (2000). Bimanual coordination and limb-specific parameterization in patients with Parkinson’s disease. Neuropsychologia.

[145] Swinnen SP, Steyvers M, van den Bergh L, Stelmach GE (2000). Motor learning and Parkinson’s disease: Refinement of within-limb and between limb coordination as a result of practice. Behavioural Brain Research.

[146] Milner TE (2002). Contribution of geometry and joint stiffness to mechanical stability of the human arm. Experimental Brain Research.

[147] Perreault EJ, Kirsch RF, Crago PE (2002). Voluntary control of static end stiffness during force regulation tasks. Journal of Neurophysiology.

[148] MacKay WA, Crammond DJ, Kwan HC, Murphy TJ (1986). Measurements of the human forearm viscoelasticity. Journal of Biomechanics.

[149] Bizzi E, Accornero N, Chapple W, Hogan N (1984). Posture control and trajectory formation during arm movement. Journal of Neuroscience.

[150] Beck BR (1998). Tibial stress injuries. An aetiological review for the purposes of guiding management. Sports Medicine (Auckland, New Zealand).

[151] Johnson GR, Dowson D, Wright V (1976). A biomechanical approach to the design of football boots. Journal of Biomechanics.

[152] Mills C, Yeadon MR, Pain MT (2010). Modifying landing mat material properties may decrease peak contact forces but increase forefoot forces in gymnastics landings. Sports Biomechanics.

[153] Kim W, Tan J, Veloso A, Vleck V, Voloshin AS (2011). The natural frequency of the foot-surface cushion during the stance phase of running. Journal of Biomechanics.

[154] Ly QH, Alaoui A, Erlicher S, Baly L (2010). Towards a footwear design tool: Influence of shoe midsole properties and ground stiffness on the impact force during running. Journal of Biomechanics.

[155] Moritz T, Farley CT (2006). Human hoppers compensate for simultaneous changes in surface compression and damping. Journal of Biomechanics.

[156] Robinovitch SN, Hayes WC, McMahon TA (1997). Distribution of contact force during impact to the hip. Annals of Biomedical Engineering.

[157] Robinovitch SN, Chiu J (1998). Surface stiffness affects impact force during a fall on the outstretched hand. Journal of Orthopaedic Research.

[158] Kang HG, Lipsitz LA (2010). Stiffness control of balance during quiet standing and dual task in older adults: The MOBILIZE Boston Study. Journal of Neurophysiology.

[159] Kuczyński M, Ostrowska B (2006). Understanding falls in osteoporosis: The viscoelastic modeling perspective. Gait Posture.

[160] Gomi H, Osu R (1998). Task dependent viscoelasticity of human multijoint arm and its spatial characteristics for interaction with environments. Journal of Neuroscience.

[161] Grasso R, Zago M, Lacquaniti F (2000). Interactions between posture and locomotion: Motor patterns in humans with bent versus with erect posture. Journal of Neurophysiology.

[162] Lacquaniti F, Carrozzo M, Borghese NA (1993). Time varying mechanical behavior of multijointed arm in man. Journal of Neurophysiology.

[163] Roth N, Wiener A, Mizrahi J (2014). Methods for dynamic characterization of the major muscles activating the lower limb joints in cycling motion. European Journal of Translational Myology: Basic and Applied Myology.

[164] Wang T, Dordevic GS, Shadmehr R (2001). Learning the dynamics of reaching movements results in the modification of arm impedance and long latency perturbation responses. Biological Cybernetics.

[165] Patla AE, Ishac MG, Winter DA (2002). Anticipatory control of center of mass and joint stability during arm movements from a standing posture: Interplay between active and passive control. Experimental Brain Research.

[166] Tsuji T, Morasso P, Sanguineti V (1997). Human arm impedance in multi-joint movements. Self organization, computational maps, and motor control.

[167] Xu Y, Hollerbach JM (1998). Identification of human joint mechanical properties from single trial data. IEEE Transactions on Biomedical Engineering.

[168] Burr DB, Holloszy JO (1997). Bone, exercise and stress fracture. Exercise and sport sciences review.

[169] Yoshikawa T, Mori S, Santiesteban AJ, Sun TC, Hafstad E, Chen J, Burr DB (1994). The effects of muscle fatigue on bone strain. Journal of Experimental Biology.

[170] Reeder MT, Dick BH, Atkins JK, Probis AB, Martinez JM (1996). Stress fractures: Current concepts of diagnosis and treatment. Sports Medicine (Auckland, New Zealand).

[171] Gittoes MJ, Kerwin DG (2009). Interactive effects of mass proportions and coupling properties on external loading in simulated forefoot impact landings. Journal of Applied Biomechanics.

[172] Liu W, Nigg BM (2000). A mechanical model to determine the influence of masses and mass distribution on the impact force during running. Journal of Biomechanics.

[173] Pain MTG, Challis JH (2002). Soft tissue motion during impacts: Their potential contribution to energy dissipation. Journal of Biomechanics.

[174] Zadpoor A, Nikooyan AA, Arshi AR (2007). A model-based parametric study of impact force during running. Journal of Biomechanics.

[175] Zadpoor A, Nikooyan AA (2010). Modeling muscle activity to study the effects of footwear on the impact forces and vibrations of the human body during running. Journal of Biomechanics.

[176] Langzam E, Nemirovsky Y, Isakov E, Mizrahi J (2006). Partition between volitional and induced forces in electrically augmented dynamic muscle contractions. IEEE Transactions on Neural Systems and Rehabilitation Engineering.

[177] Radin EL (1986). Role of muscles in protecting athletes from injury. Acta Medica Scandinavica Supplementum.

[178] Light LH, McLellan GE, Klenerman L (1980). Skeletal transients on heel strike in normal walking with different footwear. Journal of Biomechanics.

[179] Kim, W., Voloshin, A. S., Simkin, A., & Milgrom, C. E. (1990). Study of the foot performance during intensive march. In *Abstracts of the sixth international Jerusalem symposium on sport injuries*, 8–9 January (p. 40). Israel: The Hebrew University of Jerusalem.

[180] Andriacchi TP, Andersson GB, Fermier RW, Stern D, Galante JO (1980). A study of lower limb mechanics during stairclimbing. Journal of Bone and Joint Surgery.

[181] Stauber WT (1989). Eccentric action of muscles: Physiology, injury and adaptation. Exercise and Sport Sciences Reviews.

[182] Zelisko JA, Noble HB, Porter M (1982). A comparison of men’s and womens’ professional basketball injuries. The American Journal of Sports Medicine.

[183] Flynn JM, Holmes JD, Andrews DM (2004). The effect of localized leg muscle fatigue on tibial impact acceleration. Clinical Biomechanics.

[184] Wasserman K, Whipp BJ, Koyal SN, Beaver WL (1973). Anaerobic threshold and respiratory gas exchange during exercise. Journal of Applied Physiology.

[185] Whipp BJ (1987). Dynamics of pulmonary gas exchange. Circulation.

[186] Mizrahi J, Verbitsky O, Isakov E (2000). Shock accelerations and attenuation in downhill and level running. Clinical Biomechanics.

[187] Mizrahi J, Voloshin A, Russek D, Verbitsky O, Isakov E (1997). The influence of fatigue on EMG and shock absorption in running. Basic and Applied Myology.

[188] Verbitsky O, Mizrahi J, Voloshin A, Treiger J, Isakov E (1998). Shock absorption and fatigue in human running. Journal of Applied Biomechanics.

[189] Voloshin A, Mizrahi J, Verbitsky O, Isakov E (1998). Dynamic loading on the human musculoskeletal system—effect of fatigue. Clinical Biomechanics.

[190] Baker J, Frankel VH, Burstein A (1972). Fatigue fractures: Biomechanical considerations. The Journal of Bone and Joint Surgery.

[191] Nordin M, Frankel V, Nordin M, Frankel V (1989). Biomechanics of bone. Basic biomechanics of the musculoskeletal system.

[192] Mizrahi J, Verbitsky O, Isakov E (2000). Fatigue-related loading imbalance on the shank in running: A possible factor in stress fractures. Annals of Biomedical Engineering.

[193] Lafortune MA, Hennig EM, Lake MJ (1996). Dominant role of interface over knee angle for cushioning impact loading and regulating initial leg stiffness. Journal of Biomechanics.

[194] McMahon TA, Valiant G, Frederick EC (1987). Groucho running. Journal of Applied Physiology.

[195] Panjabi MM, Brand RA, White AA (1975). Three-dimensional flexibility and stiffness properties of the human thoracic spine. Journal of Biomechanics.

[196] Kurutz M (2006). In vivo age- and sex-related creep of human lumbar motion segments and discs in pure centric tension. Journal of Biomechanics.

[197] Lebiedowska MK, Fisk JR (1999). Passive dynamics of the knee joint in healthy children and children affected by spastic paresis. Clinical Biomechanics.

[198] Oka H, Yamamoto T (1987). Dependence of biomechanical impedance upon living body structure. Medical and Biological Engineering and Computing.

[199] Hardt DF (1978). Determining muscle forces in the leg during normal human gait: An application and evaluation of optimization methods. Journal of Biomechanical Engineering: Transactions of the ASME.

[200] Zajac, F. E. (1988). Muscle and tendon: Properties, models, scaling and application to biomechanics and motor control. In *Critical reviews in biomedical engineering*. Boca Raton, FL: CRC Press. 2676342

[201] Zahalak GI, Winters JM, Woo SLY (1990). Modeling muscle mechanics (and energetics). Multiple muscle systems.

[202] Winters J, Winters JM, Woo SLW (1990). Hill-based muscle models: A systems engineering perspective. Multiple muscle systems.

[203] Close RI (1972). Dynamic properties of mammalian skeletal muscles. Physiological Reviews.

[204] Gordon AM, Huxley AF, Julian FJ (1966). The variation in isometric tension with sarcomere length in vertebrate muscle fibers. Journal of Physiology (London).

[205] Ramzey RW, Street SF (1940). The isometric length–tension diagram of isolated skeletal muscle fibers of the frog. Journal of Cellular and Computational Physiology.

[206] Woledge, R. C., Curtin, N. A., & Homsher, E. (1985). Energetic aspects of muscle contraction. In *Monographs of the Physiological Society* No. 41. London: Academic, Harcourt Brace Jovanovich. 3843415

[207] Huxley AF, Simmons RM (1971). Proposed mechanism of force generation in striated muscle. Nature.

[208] Stephenson DG, Williams DA (1982). Effects of sarcomere length on the force–pCa relation in fast- and slow-twitch skinned muscle fiber from the rat. Journal of Physiology (Cambridge).

[209] Bahler AS (1967). Series elastic component of mammalian skeletal muscle. American Journal of Physiology.

[210] Woledge RC (1961). The thermoelastic effect of change of tension in active muscle. Journal of Physiology.

[211] Cannavan D, Coleman DR, Blazevich AJ (2012). Lack of effect of moderate-duration static stretching on plantar flexor force production and series compliance. Clinical Biomechanics.

[212] Giat Y, Mizrahi J, Levine WS, Chen J (1994). Simulation of distal tendon transfer of the biceps brachii and the brachialis muscles. Journal of Biomechanics.

[213] Lan N, Crago PE (1994). Optimal control of antagonistic muscle stiffness during voluntary movements. Biological Cybernetics.

[214] Meimoun Y, Mizrahi J (2000). Biomechanical simulation of an amputated forearm with and without prosthesis. The Proceedings of the Institution of Mechanical Engineers, Part H: Journal of Engineering in Medicine.

[215] Winters JM, Kleweno DG (1993). Effect of initial upper-limb alignment on muscle contributions to isometric strength curves. Journal of Biomechanics.

[216] Giat Y, Mizrahi J, Levy M (1993). A musculo-tendon model of the fatigue profiles of paralyzed quadriceps muscle under FES. IEEE Transactions on Biomedical Engineering.

[217] Giat Y, Mizrahi J, Levy M (1996). A model of fatigue and recovery in paraplegic’s quadriceps muscle when subjected to intermittent stimulation. Journal of Biomechanical Engineering: Transactions of the ASME.

[218] Levin O, Mizrahi J (1999). EMG and metabolic-based prediction of force in paralyzed quadriceps muscle under interrupted simulation. IEEE Transactions on Rehabilitation Engineering.

[219] Levin O, Mizrahi J, Isakov E (2000). Transcutaneous FES of paralyzed quadriceps: Is knee torque affected by unintended activation of the hamstrings?. Journal of Electromyography and Kinesiology.

[220] Levy M, Mizrahi J, Susak Z (1990). Recruitment, force and fatigue characteristics of quadriceps muscles of paraplegics isometrically activated by surface functional stimulation. Journal of Biomedical Engineering.

[221] Levy M, Kushnir T, Mizrahi J, ltzchak Y (1993). *In vivo* 31P NMR studies of paraplegics’ muscles activated by functional electrical stimulation. Magnetic Resonance in Medicine.

